# Profiling cases of button battery ingestion using Canadian and British Columbia poison centre data

**DOI:** 10.24095/hpcdp.46.1.05

**Published:** 2026-01

**Authors:** Jeffrey Trieu, Neil Arason, Mojgan Karbakhsh, David A. McVea

**Affiliations:** 1 Environmental Health Services, BC Centre for Disease Control, Vancouver, British Columbia, Canada; 2 Island Health Authority, Victoria, British Columbia, Canada; 3 BC Injury Research and Prevention Unit, BC Children’s Hospital Research Institute, Vancouver, British Columbia, Canada; 4 School of Population and Public Health, University of British Columbia, Vancouver, British Columbia, Canada

**Keywords:** chemical burns, accident prevention, surveillance, poison control centers

## Abstract

Ingestion of button batteries poses an acute life-threatening injury risk, particularly for small children. The Canadian Surveillance System for Poison Information reported 1021 single-substance button-battery ingestion cases from 2020 to 2023, and the British Columbia Drug and Poison Information Centre (DPIC) managed 548 unintentional ingestion cases from 2013 to 2023. Nearly all the DPIC cases required hospital admission for X-ray imaging, and seven patients required surgical removal of the battery from the esophagus. Our findings support developing product warning labels and enforcing child-resistant battery packaging and compartments on consumer products.

**Highlights**

- 62% of Canadian Surveillance System for Poison Information (CSSPI) cases and 69% of British Columbia Drug and Poison Information Centre (DPIC) cases of ingested button batteries occurred among children younger than 6 years.
- 13% of CSSPI cases and 15% of DPIC cases were among adults older than 59 years. In nearly all these DPIC cases, hearing aid batteries were ingested.
- 91% of DPIC cases were referred to hospital or were already there at the time of the call to DPIC.
- The battery had to be surgically removed from the esophagus of 7 individuals.

## Introduction

Button batteries, also known as coin or disk batteries, are single-cell batteries commonly found in small electronic products. They are shaped like a flat cylinder and are typically 5 to 25 mm in diameter and 1 to 6 mm in height. Given their small size, these batteries can be easily ingested, including by infants and small children. The battery can pass uneventfully through the digestive tract, but there is a risk that it lodges in the esophagus, which can cause a pressure wound or alkaline chemical burn. Severe injury can start within 2 hours of ingestion and can include esophageal perforations, fistulas and related complications.^1^,^2^ In short, ingesting button batteries poses an acute life-threatening injury risk and requires emergency imaging and endoscopic removal if the battery is lodged.

Surveillance of button battery–related injuries in Canada is limited. The United States *International Classification of Diseases, Tenth Revision, Clinical Modification* (ICD-10-CM) has a code for these events (W44A.A1), but the Canadian modification, ICD-10-CA, has no similar code.^3^ The Consumer Product Safety Program collates health and safety incidents that companies are mandated to report and incidents that consumers and other involved parties elect to report. The Program reported that two children, one in 2021 and the other in 2022, died after ingesting a button battery.^4^ From 2006 to 2020, the Canadian Hospitals Injury Reporting and Prevention Program reported an increase in the number of button battery–related visits to emergency departments at their sentinel hospital sites.^5^,^6^ A 2022 Canadian Paediatric Surveillance Program (CPSP) survey of 1067 pediatric physicians found that they cumulatively managed 815 button battery ingestions in the previous 12 months, with 77 patients requiring endoscopic removal of the batteries.^7^

Poison centres are a valuable data source for battery-related injuries but, to our understanding, these cases have not been reported. In this article, we describe cases of button battery ingestions managed by the British Columbia Drug and Poison Information Centre (DPIC) or reported by the Canadian Surveillance System for Poison Information (CSSPI).

## Methods

Poison centres are 24-7 telephone services that provide toxicological and clinical guidance to health care professionals and the public. Clinicians at poison centres follow cases as necessary to acute resolution, compile relevant clinical information and document the event in free-text chart notes and data fields standardized by America’s Poison Centers (APC). CSSPI collates data from the five poison centres that serve all of Canada, including those from DPIC, whose jurisdiction covers British Columbia and Yukon.^8^

In this study, we examined ingestion cases with a button battery–related APC substance code. There are eight in total, each related to a different formulation (e.g. lithium) and identified by a 265 prefix. DPIC cases were available from 2013 to 2023. CSSPI cases were limited to single-substance cases and available from 2020 to 2023.

Given our access to DPIC chart notes through Environmental Health Services at the BC Centre for Disease Control, we could include DPIC cases without a button battery APC code but with a related free-text description. We reviewed the chart notes of all DPIC cases to confirm inclusion and also extracted information on the exposure and treatment course and the battery size, type and source. We conducted a descriptive analysis of the standardized data fields and the information extracted from the chart notes. We used population estimates to compute annual per capita case rates, 2021 census population values to compute person-year rates and all DPIC or CSSPI exposure case counts to compute crude rates of all exposure cases.^9^,^10^

We completed all analyses in R version 4.3.2 (R Foundation for Statistical Computing, Vienna, AT).^11^

This project is exempt from research ethics board review because the BC Centre for Disease Control, where DPIC is housed, is mandated to conduct surveillance analyses and report deidentified data by the British Columbia *Public Health Act.*^12^

## Results

From 2013 to 2023, DPIC managed 571 cases of button battery ingestion. Of these, 566 (99%) involved ingestion of only a battery (i.e. a single-substance case) and five pediatric cases involved simultaneous ingestion of medications (n = 2), magnets (n = 2) and a household cleaning product (n = 1). From 2020 to 2023, CSSPI reported 1021 single-substance button battery ingestion cases. 

About two-thirds of cases—394 (69%) of DPIC cases and 634 (62%) of CSSPI cases—were among children younger than 6 years (Table 1). Of the DPIC cases, 311 were infants and toddlers younger than 3years. Among cases in individuals older than 5 years, the highest per capita case rate was among adults older than 59 years. There were more males than females overall. Among DPIC cases, this sex difference is seen in children and youth aged younger than 20 years. 

**Table 1:** Age- and sex-stratified counts and rates of cases of button battery ingestion

Age, years	Total case count, n	Total case rate		Case count by sex, n			Case rate by sex per 100 000 person-years	
		Per 10 000 all exposure cases	Per 100 000 person-years	Females		Males	Females	Males
DPIC cases^b^								
All ages	571	18.49	1.03	271	296		0.96	1.09
< 6 years	394	33.76	13.4	181	211		12.63	14.00
6–19 years	52	13.13	0.66	18	33		0.48	0.81
20–59 years	14	1.89	0.05	9	5		0.06	0.03
> 59 years	88	33.55	0.58	47	41		0.58	0.57
Unknown	23	4.41	n/a	16	6		n/a	n/a
CSSPI cases^c^								
All ages	1021	13.52	0.69	481	536		0.64	0.74
< 6 years	634	26.09	7.10	n/a	n/a		n/a	n/a
6–19 years	152	12.69	0.66	n/a	n/a		n/a	n/a
20–59 years	73	2.83	0.09	n/a	n/a		n/a	n/a
> 59 years	135	16.61	0.35	n/a	n/a		n/a	n/a
Unknown	27	5.06	n/a	n/a	n/a		n/a	n/a

**Abbreviations: **CSSPI, Canadian Surveillance System for Poison Information; DPIC, British Columbia Drug and Poison Information Centre; n/a, not available. 

^a^ Four DPIC and four CSSPI cases with unknown sex are not included in sex-stratified counts and rates. 

^b^ Managed at the DPIC from 2013 to 2023. 

^c^ Reported to CSSPI from 2020 to 2023. CSSPI age- and sex-stratified counts are unknown. 

**Abbreviations: **CSSPI, Canadian Surveillance System for Poison Information; DPIC, British Columbia Drug and Poison Information Centre; n/a, not available. 

^a^ Four DPIC and four CSSPI cases with unknown sex are not included in sex-stratified counts and rates. 

^b^ Managed at the DPIC from 2013 to 2023. 

^c^ Reported to CSSPI from 2020 to 2023. CSSPI age- and sex-stratified counts are unknown. 

Nearly all DPIC cases were unintentional (n = 548; 96%). There were 21 intentional ingestion cases and two with unknown intentionality. Most calls to DPIC (n=432; 76%) were from family members or friends of the person who ingested the battery (referred to as “the patient”), 78 (14%) were from healthcare professionals and 44 (8%) were from the patients themselves; in 28 cases (5%), the callers were unknown (data not shown).

**Figure 1:**
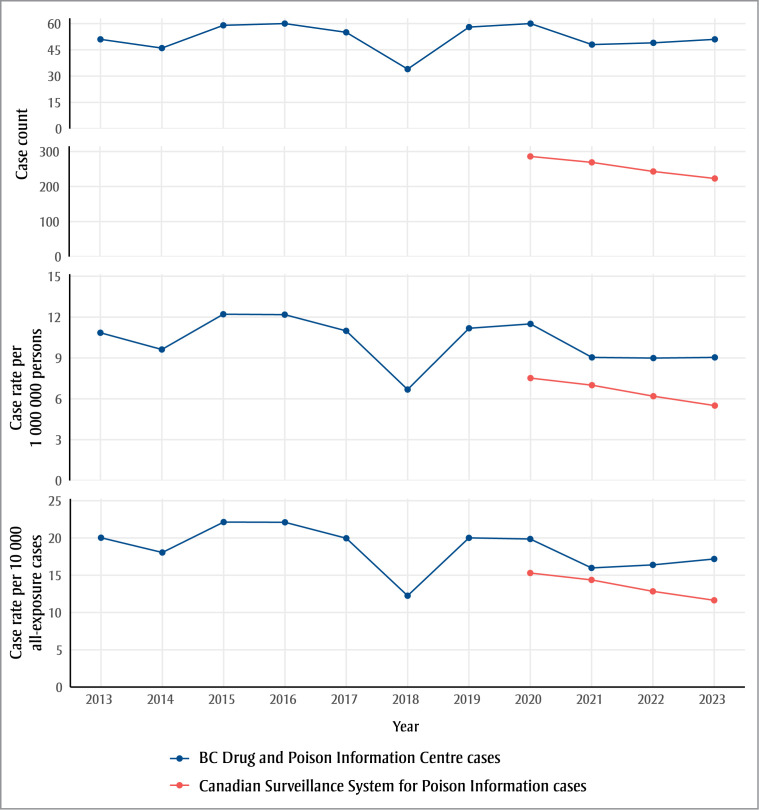
Annual counts and rates of cases of button battery ingestion

^a^ Cases managed at the British Columbia Drug and Poison Information Centre (DPIC) from 2013 to 2023 and single-substance button-battery ingestion cases collated by the Canadian Surveillance System for Poison Information (CSSPI) from 2020 to 2023.

Other than a decrease in 2018, the annual trend of DPIC cases was stable from 2013 to 2020 (Figure 1). The counts and rates decreased slightly in 2021 and remained stable through 2023. There was a consistent annual decrease in counts and rates of cases reported to CSSPI.

We found no information on the battery source (n = 281; 49%), type (n=464; 81%) or size (n = 504; 88%) in most cases. When such information was available, we found that most of the batteries were from hearing aids (n = 131), toys (n= 52), watches (n = 47), flashlights (n=23) and miscellaneous household items (n = 37). In 79 of 88 cases of adults older than 59 years, the battery was from a hearing aid. The patient mistook the battery for a pill in 31 cases. A medical condition such as dementia was implicated in 13 cases. 

A total of 176 CSSPI cases (17%) and 49 DPIC cases (9%) were not referred to hospital. In 42 of the DPIC cases, the battery was excreted via stool, spat out or found (confirming noningestion) at the time of call. In seven cases of adults older than 59 years who had ingested smaller batteries, the individual was advised to monitor their stool and symptoms at home. 

At the time of the call, 810 CSSPI cases (79%) were referred to hospital, were already there or were on their way. The patient flow was unknown in 35 CSSPI cases (3%). In total, 495 DPIC cases (87%) were referred to hospital and 27patients had already been seen in hospital when DPIC was called. The specific outcome was unknown in 363 cases.

X-ray imaging showed the battery to be past the esophagus in 65 cases; it was not found by X-ray in 78 cases and passed via stool, vomited or found (confirming noningestion) in nine cases. The battery needed to be surgically removed from the esophagus in seven cases. In two cases of the battery being excreted or vomited, the patient was left with an ulceration or notable abrasion of their esophagus.

## Discussion

This analysis of calls to poison centres contributes to the limited understanding of button battery–related injuries in Canada. Compiling data from the five Canadian poison centres, CSSPI reported 0.69 single-substance button-battery ingestion cases per 100 000 person-years from 2020 to 2023. DPIC, the poison centre that serves British Columbia and Yukon, had a higher rate of cases, 1.03 per 100000 person-years from 2013 to 2023. This difference should be corroborated with other data sources. Although surgical removal of the battery was not common (n = 7), nearly all imposed a health system burden. DPIC did not follow 363 cases after referral to hospital or 65 cases after the battery was found in the esophagus. As such, it is likely that serious outcomes were undercounted.

There are limitations with these data. Poison centres do not manage all battery-related injuries and the reported statistics do not represent true population incidence. For example, physicians surveyed by the CPSP in 2022 reported 815 button battery ingestions in just one year,^7^ whereas CSSPI reported between 223 and 286 cases each year from 2020 to 2023. 

Poison centres provide acute clinical guidance and do not capture long-term complications, even after surgical removal of batteries.^13^ Battery type and size are largely unknown, which precludes analyses with these product factors. The device source was also unknown in approximately half the cases. When this was known, nearly all cases in adults older than 59 years involved hearing aid batteries. This suggests an opportunity for targeted education.

## Conclusion

The *Canada Consumer Product Safety Act* regulates consumer products in a postmarket regulatory regimen.^14^ At the time of writing, products containing button batteries have been identified as a “hazard of concern” under this statute, meaning that Health Canada is considering further risk management actions. Our findings support following the regulatory path in Australia, many European nations and the United States in enforcing child-resistant packaging of button batteries, child-resistant compartments on consumer products that use button batteries and product warning labels and establishing recall provisions.^15^-^17^ Given that industry must follow other countries’ regulations in a global marketplace, it is reasonable to implement and enforce the same consumer product regulations in Canada.

We also recommend that the ICD-10-CA adopt and promote the use of the button battery ingestion/insertion code to support surveillance efforts with hospitalization and mortality records.

## Acknowledgements

The authors would like to thank the clinical staff at the British Columbia DPIC and all other Canadian poison centres for considering the surveillance and analytic outputs of their work beyond their clinical service; the members of the provincial and national working group for guiding this work; and CSSPI, led by Health Canada, and Canadian poison centres, for collating and providing pan-Canadian poison centre data.

## Funding

None.

## Conflicts of interest

The authors have no conflicts of interest to declare.

## Authors’ contributions and statement

JT: Formal analysis, writing—original draft. 

NA: Conceptualization, investigation, writing—review and editing. 

MK: Conceptualization, investigation, writing—review and editing. 

DM: Supervision, writing—review and editing. 

All the authors reviewed and approved the final version of the manuscript.

The content and views expressed in this article are those of the authors and do not necessarily reflect those of Health Canada or the Government of Canada.
